# Single-nucleus RNA velocity reveals critical synaptic and cell-cycle dysregulations in neuropathologically confirmed Alzheimer’s disease

**DOI:** 10.1038/s41598-024-57918-x

**Published:** 2024-03-27

**Authors:** Quadri Adewale, Ahmed F. Khan, David A. Bennett, Yasser Iturria-Medina

**Affiliations:** 1 Neurology and Neurosurgery Department, Montreal Neurological Institute, McGill University, Y I-M, 3801 University Street, Room NW312, Montreal, H3A 2B4 Canada; 2 McConnell Brain Imaging Centre, Montreal Neurological Institute, McGill University, Montreal, Canada; 3 Ludmer Centre for Neuroinformatics and Mental Health, McGill University, Montreal, Canada; 4 Rush Alzheimer’s Disease Center, Rush University Medical Center, Chicago, IL USA; 5 Department of Neurological Sciences, Rush University Medical Center, Chicago, IL USA

**Keywords:** RNA velocity, Single-cell RNA-seq, Neuropathological Alzheimer’s disease, Synapse, Cell development, Alzheimer's disease, Genetics of the nervous system

## Abstract

Typical differential single-nucleus gene expression (snRNA-seq) analyses in Alzheimer’s disease (AD) provide fixed snapshots of cellular alterations, making the accurate detection of temporal cell changes challenging. To characterize the dynamic cellular and transcriptomic differences in AD neuropathology, we apply the novel concept of RNA velocity to the study of single-nucleus RNA from the cortex of 60 subjects with varied levels of AD pathology. RNA velocity captures the rate of change of gene expression by comparing intronic and exonic sequence counts. We performed differential analyses to find the significant genes driving both cell type-specific RNA velocity and expression differences in AD, extensively compared these two transcriptomic metrics, and clarified their associations with multiple neuropathologic traits. The results were cross-validated in an independent dataset. Comparison of AD pathology-associated RNA velocity with parallel gene expression differences reveals sets of genes and molecular pathways that underlie the dynamic and static regimes of cell type-specific dysregulations underlying the disease. Differential RNA velocity and its linked progressive neuropathology point to significant dysregulations in synaptic organization and cell development across cell types. Notably, most of the genes underlying this synaptic dysregulation showed increased RNA velocity in AD subjects compared to controls. Accelerated cell changes were also observed in the AD subjects, suggesting that the precocious depletion of precursor cell pools might be associated with neurodegeneration. Overall, this study uncovers active molecular drivers of the spatiotemporal alterations in AD and offers novel insights towards gene- and cell-centric therapeutic strategies accounting for dynamic cell perturbations and synaptic disruptions.

## Introduction

Recent advances in single-nucleus RNA sequencing (snRNA-seq) have provided an unprecedented ability to disentangle cellular-level transcriptomic alterations and heterogeneity in Alzheimer’s disease (AD)^1,2^. Early differential expression with neuropathological AD progression have been found to be cell-type dependent while apparent upregulation of genes in the later stages are shared across cell types, suggesting that transcriptional responses to disease are highly driven by cell states^1^. Furthermore, cell type-specific analysis has revealed the molecular signatures of preferentially affected cell populations, demonstrating that morphology alone cannot sufficiently determine cell type vulnerability in pathologic AD^3–5^.

Nevertheless, static snRNA-seq abundance provides only fixed snapshots of cellular states, not revealing temporal dynamics of genes at individual cells^5^. The recently proposed rate of change of mRNA, otherwise known as RNA velocity (RNA-vel)^6^, provides a novel method to capture temporal dynamics in mRNA abundance by comparing spliced and unspliced mRNA counts. In the initial model, ratio of intronic to exonic sequence counts in constant (steady-state) transcription is obtained, and RNA-vel is estimated as the deviation or residual of this ratio from the expected steady-state ratio^6^. Further methods have been developed to capture (potentially) unobserved steady states and gene stochasticity^5^. Positive and negative RNA velocities imply upregulation and downregulation of a gene, respectively. Notably, RNA-vel analysis was used to infer developmental trajectories of healthy cells^7,8^ and unravel pathological changes in cancer cells^9^. This paradigm shift from descriptive to predictive RNA modelling is offering a deeper understanding of complex cell-level processes in health and disease, with promising implications to improve treatment strategies for multiple disorders.

In the context of the progression of neuropathology, it is unclear whether genes that are differentially expressed across disease states are also the genes driving evolution and vulnerability of diseased cells. Genes with different RNA velocity values might better capture or capture complementary aspects of the time-resolved molecular dysregulations and prodromal differences underlying neurodegeneration. Here, we extend previous single-nucleus RNA (snRNA) analysis in AD in three fundamental ways. First, we use postmortem snRNA-seq data from the prefrontal cortex of subjects with varied levels of AD pathology (N = 48) to identify cell type-specific RNA velocity differences associated with neuropathology. Second, we demonstrate that dynamically altered genes in AD pathology, i.e., genes with differential RNA velocities, are qualitatively different from the genes showing differential expression patterns. Third, we reproduce the main observed cell type-specific RNA velocity differences in an independent pathologic AD sample. Overall, our results highlight the critical importance of further considering dynamic single-cell molecular processes underlying AD progression as opposed to only its static cellular RNA mechanisms.

## Results

### Data origin and single-nucleus RNA velocity estimation

**Figure 1:**
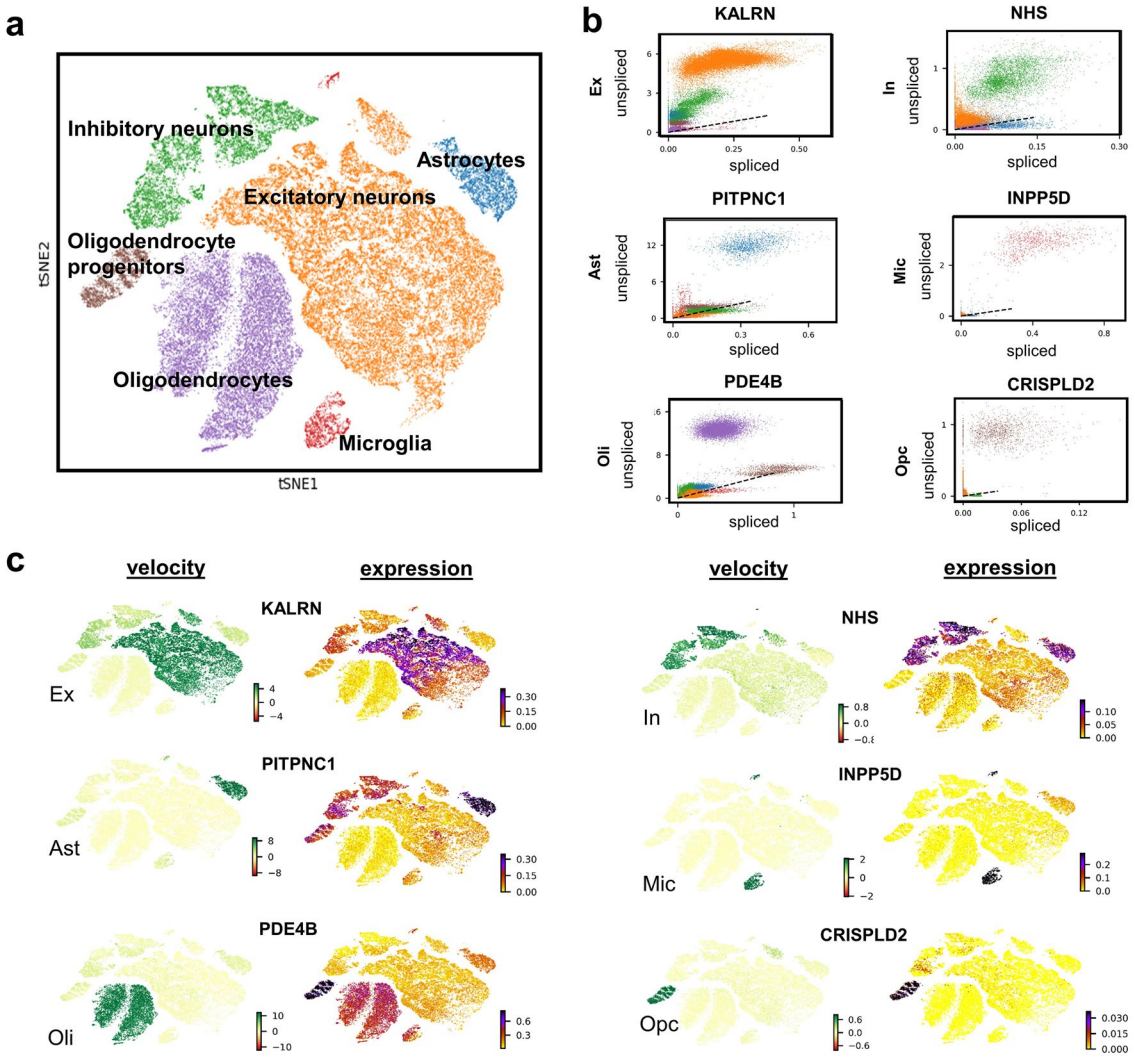
Single-nucleus RNA-seq of the prefrontal cortex of 48 individuals across the Alzheimer’s disease (AD) spectrum. (**a**) t-SNE visualization of clusters are annotated by cell type (excitatory neurons, inhibitory neurons, astrocytes, microglia, oligodendrocytes, and oligodendrocyte progenitor cells). (**b**) Relationship between unspliced and spliced mRNA counts of genes driving differential dynamics of each cell type. The dashed black line represents the estimated steady state ratio of the unspliced to spliced mRNA. RNA velocity is obtained as the residual of the observed intronic to exonic RNA ratio from this steady state line. (**c**) RNA velocity and expression patterns of the dynamic genes. Larger variation in velocity is driven by transcriptional dynamics.

Single nucleus RNA-seq data was obtained from the prefrontal cortex of 48 postmortem human brain samples^1^ (Sects. “Methods”, “Dataset-1”). Twenty-four of these individuals had no or low β-amyloid burden or other pathologies (control). The remaining twenty-four presented mild to severe AD-pathology (amyloid burden, neurofibrillary tangles, global pathology, and cognitive impairment). After pre-processing, 65,422 snRNA-seq profiles with 16,844 transcripts (corresponding to 16,829 unique genes) were obtained. A predefined cluster list^1^ was used to annotate and assign the cells to six different types: excitatory neurons, inhibitory neurons, astrocytes, microglia, oligodendrocytes, and oligodendrocyte progenitor cells (Fig. 1a).

Single-nucleus RNA velocities (snRNA-vel) of all genes across each cell type in the 48 subjects were calculated using a probabilistic model^5^ (Sect. “Methods”, “RNA velocity estimation”). This probabilistic (stochastic) method for RNA-vel estimation is preferred over the originally proposed steady-state model^6^ since the former largely accounts for cell heterogeneity and differential kinetics, while achieving higher computational efficiency^5^. To identify the genes that may help explain the velocity vector fields across the six types, we selected the top genes that show cell type-specific differential transcriptional dynamics (Sect. “Methods”, “RNA velocity estimation”). As shown in Fig. 1b, the dependency between unspliced and spliced mRNA counts of the genes gives the expected cell type-specific velocity values depicted as the residual from the dotted line (representing the constant transcriptional state). We then projected the expression and velocity values of these top genes to t-SNE space (Fig. 1c). We observed more variation and cell type-specificity in RNA velocity compared to gene expression, suggesting that the velocity estimations are largely driven by transcriptional dynamics rather than gene expression (Fig. 1c). For example, *PDE4B* exhibits an oligodendrocyte-specific dynamics even though its expression is spread across different cell types.

To demonstrate the suitability of using single nuclei for RNA velocity, we first compared both nucleus-derived (snRNA-seq) and cell-derived (scRNA-seq) RNA velocities in the microglia of the same subject. Notably, we observed (Fig. S1) strong correlations (ranging from 0.94 to 0.99) between the velocity estimates of the snRNA-seq and scRNA-seq, supporting the precision of snRNA-seq for RNA velocity calculation (see Fig. S1). Interestingly, the variations observed in velocity correlations from any random pair of single cells are comparable to the variations in velocity correlations between any single cell and single nucleus. A previous study also found concordant RNA velocity estimates between matched single nuclei and single cells from rabbit retina^10^. Finally, we recalculated RNA velocity using veloVI^11^, another method of RNA velocity estimation based on deep generative modeling. Comparison of the results from scVelo and veloVI shows similar velocity estimates and trajectory inference (Fig. S2).

### Static vs dynamic genetic-cell modifications underlying AD evolution

**Figure 2:**
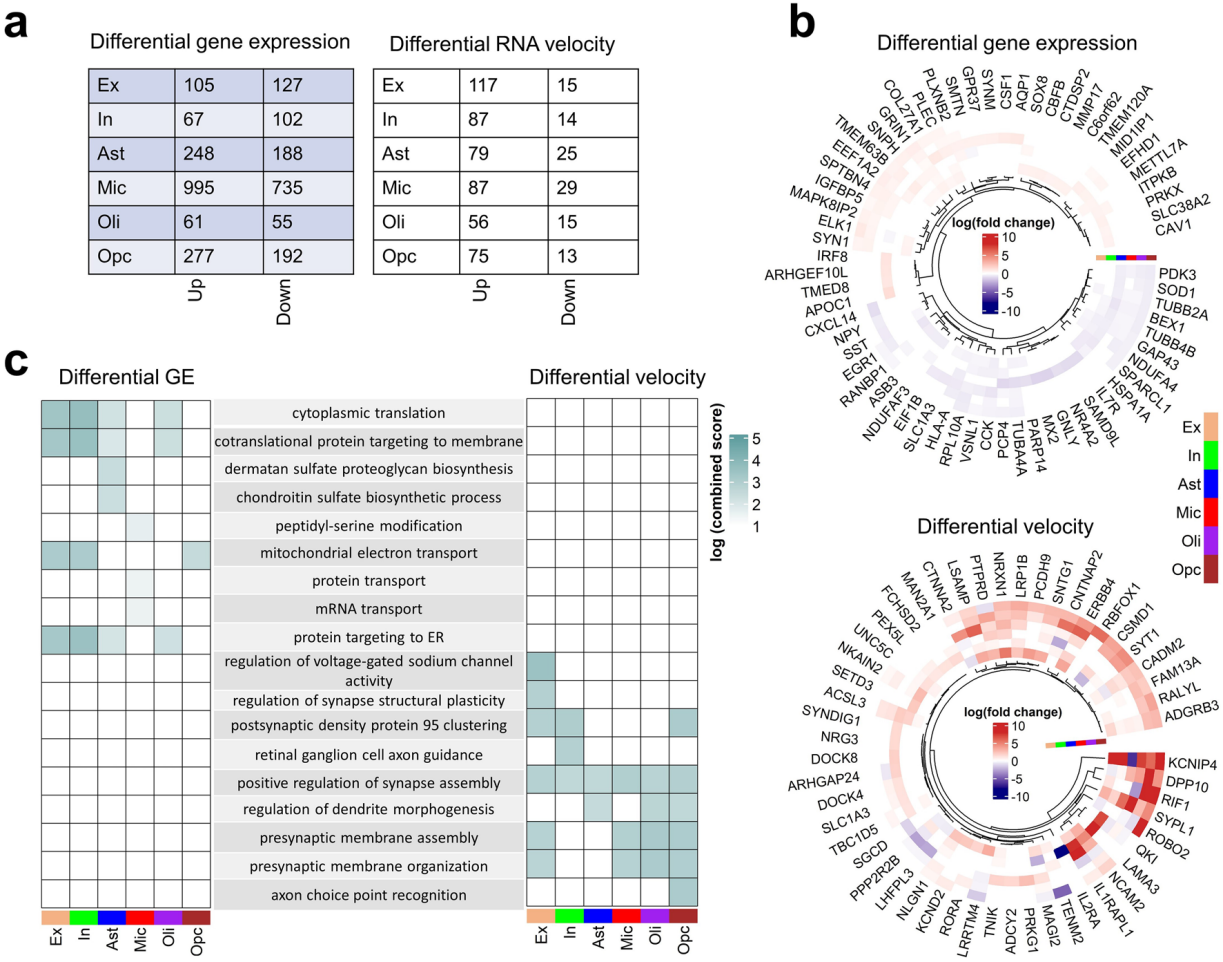
Differences in RNA velocity and gene expression underlying neuropathological AD progression. (**a**) Number of genes with differential expression patterns and velocity between controls (n = 24) and AD subjects (n = 24) across cell types (two-sided Wilcoxon rank-sum test, permutation-based FDR-corrected q-value < 0.05, log_2_ (mean gene expression or velocity AD/mean gene expression or velocity in control) > 0.25 or <  − 0.25). (**b**) Cell-type specific changes (log_2_ (fold change)) for the top genes with differential expression (DE) (top) and differential velocity (bottom) between control and AD subjects. (**c**) Comparison of biological pathways associated with differential expression and differential velocity.

We sought to investigate if there are global AD-pathology dependent differences in RNA velocity patterns across cell types. We evaluated the cell type-specific differences in RNA velocity between the control and AD-pathology subjects using Wilcoxon rank-sum test. We then compared the results with those obtained for differential gene expression. Across all cell types, we observed lesser genes with differential RNA velocity (612) than those with differential expression (3152) (Fig. 2a, Supplementary Table 1). The top ranked genes underlying RNA expression and velocity variations are presented in Fig. 2b. Furthermore, higher fold changes were observed for RNA velocities compared to gene expression. To exclude the possible confounding impact of age on the observed differential transcriptional kinetics, we reanalyzed the group differences in RNA velocity after correcting for age. The new result was consistent with the original finding without correction, probably because age was matched between groups (Fig. S3). Lastly, due to potential over-representation of long unspliced mRNA transcripts in neurons^12^, we checked if the differential velocity observed between the two groups may be biased by gene length. We found no correlation (R = 0.00081; *p*-value = 0.92) between the U-statistic of Wilcoxon rank-sum test and the length of genes in inhibitory neurons (Fig. S4).

Notably, 63 of the 3152 (2%) differentially expressed genes were also found to exhibit differential velocity, suggesting substantial AD-pathology related differences between these two RNA descriptors. On the one hand, the genes with only snRNA-vel differences relate to cell developmental and synaptic processes such as morphogenesis, axonal guidance, ion channel activity, synapse organization and cell assembly (Fig. 2c, Supplementary Table 2). The protein–protein interaction network of genes associated with ion channel activity and synapse organization in excitatory neurons is shown in Fig. S5. Conversely, genes with only differential expression are majorly associated with mitochondrial activity, ribosomal processes, and protein sorting. This mismatch between snRNA-vel and RNA abundance dysregulations suggests that analyzing RNA velocity provides relevant complementary information about the multifactorial molecular processes associated with neuropathological AD advance compared to differential expression.

We next investigated if the changes in RNA velocity depend on disease stage. We subgrouped the AD-pathology subjects into early- and late-AD, based on previous study^1^. Briefly, the two pathological subgroups were obtained by clustering the subjects on several clinico-pathological features^1^. Consequently, early-AD corresponds to some amyloid load with moderate neurofibrillary tangles and cognitive deficit. Late-AD subjects display higher amyloid load and increased neurofibrillary tangles, global pathology, and cognitive deficit. Comparison of the control and early-AD subjects revealed broad-scale changes in transcriptional kinetics between these subgroups (Supplementary Table 3). Corresponding analysis between control and late-AD subjects showed an increased number of affected genes in microglia, astrocytes, and oligodendrocytes, suggesting a progressive immune dysregulation (Fig. S6). However, we did not observe any notable shift in cell-type specificity of the velocity differences with disease progression.

Finally, to discern sex-dimorphic differences in transcriptional dynamics, we recomputed differential RNA velocity between the control and AD-pathology subjects while stratifying the data by sex. We found more genes with differential dynamics in females compared to males across cell types (Supplementary Table 4). Interestingly, previous single-cell studies of differential gene expression also observed more dysregulation in female subjects^1,13^, which may account for the higher disease burden in females. Nevertheless, the implicated pathways between both sexes are qualitatively similar, except for some biological processes such as lymphocyte activation and vascular process which are pronounced in the microglia of male (Fig. S7).

### Several RNA-velocity differences underlie AD neuropathological severity

**Figure 3:**
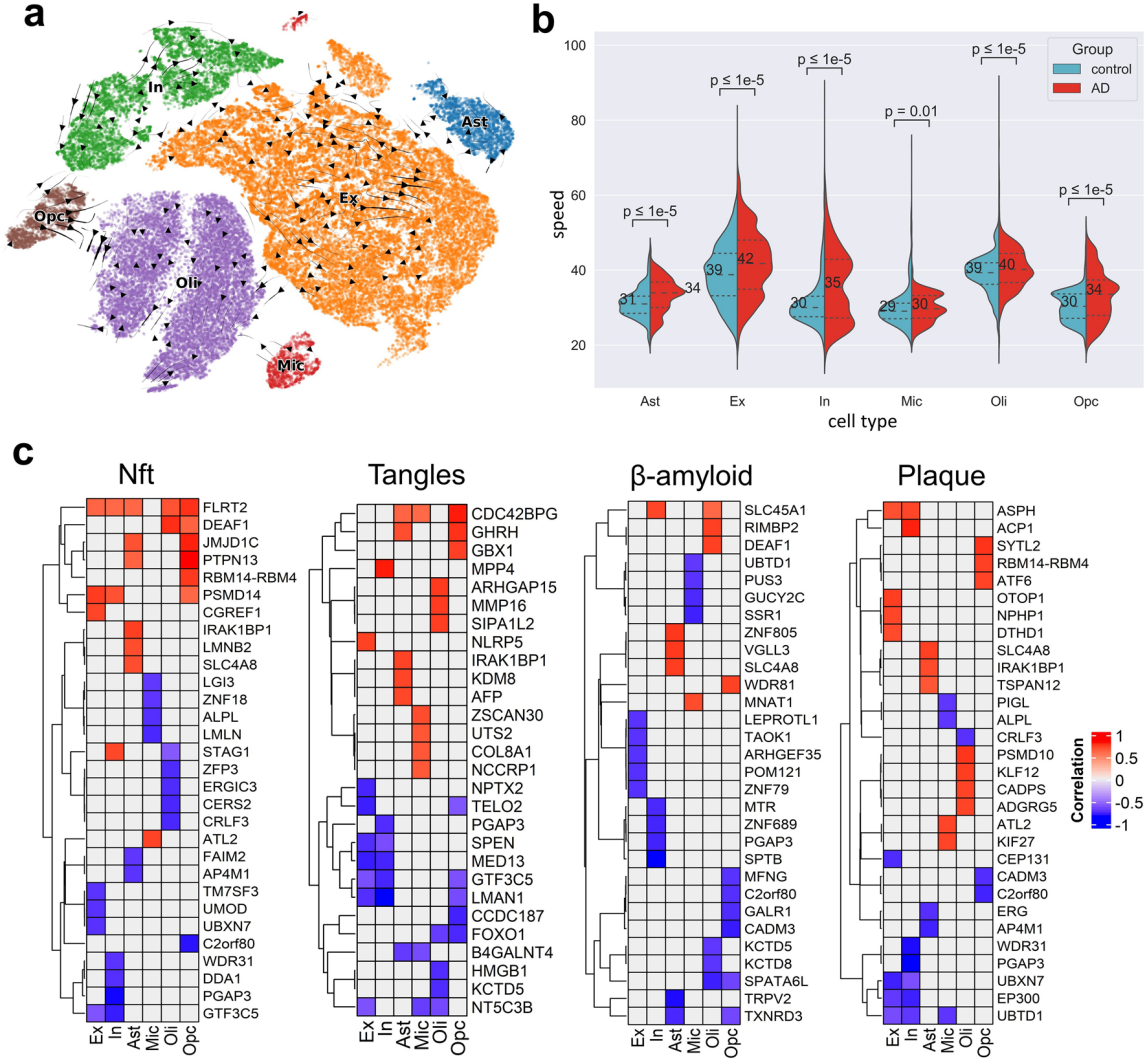
Association of RNA velocity with relevant Alzheimer’s-related neuropathological traits. (**a**) Residual velocity fields between the control and AD cells (z-score > 1.96 or < − 1.96). (**b**) Variation of cell speed between the control and AD groups. Median speed overlays plots as numbers (Wilcoxon rank-sum test *p*-value < 0.05). (**c**) Spearman’s correlation of RNA velocity with four AD-neuropathological traits: neuritic plaque count, neuronal neurofibrillary tangle (NFT) counts, overall β-amyloid load (β-amyloid) and PHF tau tangle density (tangles). The confounding effects of age, sex, education, and postmortem interval were accounted for via partial correlation. The plots show the top significant genes at FWE-corrected *p*-value < 0.001.

We proceeded to conceptualize the observed differences in RNA velocities between the control and AD groups. We projected the velocity vectors into t-SNE space and evaluated the group difference in velocity fields across all cells (Sect. "Methods", "Cell speed and residual velocity estimation"). Figure 3a shows the residual velocity fields which account for the difference between the two groups. It should be noted that the directions of the residual fields do not have any perceived biological connotation. To further understand the biological implication of the observed residual velocity fields, we calculated the speed of individual cells using the velocities across all genes. Indeed, we found a higher speed in the AD groups compared to controls, suggesting that accelerated cell changes are associated with AD neuropathology (Fig. 3b).

Lastly, we investigated the association of the RNA velocity differences with four well-known AD pathological traits: neuritic plaque (NP) and neuronal neurofibrillary tangle (NFT) counts based on histochemistry silver stain, and overall β-amyloid load (β-amyloid) and PHF tau tangle density (tangles) based on molecularly specific immunohistochemistry. We calculated Spearman’s correlation between RNA velocity of genes from the 24 AD subjects and the four neuropathological traits while adjusting for the covariates age, sex, education, and postmortem interval. The velocity-phenotype correlations of the top significant genes (FWE-corrected, *p*-value < 0.001) are shown in Fig. 3c. The genes underlying the different neuropathological traits are largely specific to cell types (Fig. S8). Only excitatory and inhibitory neurons presented a relatively high overlap (up to 13%) in significant genes associated with the different AD neuropathological traits. The other cell types substantially differed in gene-specific changes across the phenotypes. Interestingly, we found some AD-relevant genes, including *ADAM10*, associated with tangle burden in excitatory and inhibitory neurons. Some other previously reported AD genes identified include amyloid beta precursor protein binding family members, matrix metallopeptidases, notch, low-density lipoproteins, and protein kinase C’s (Supplementary Table 5).

### Cross-study validation of differential RNA velocity

**Figure 4:**
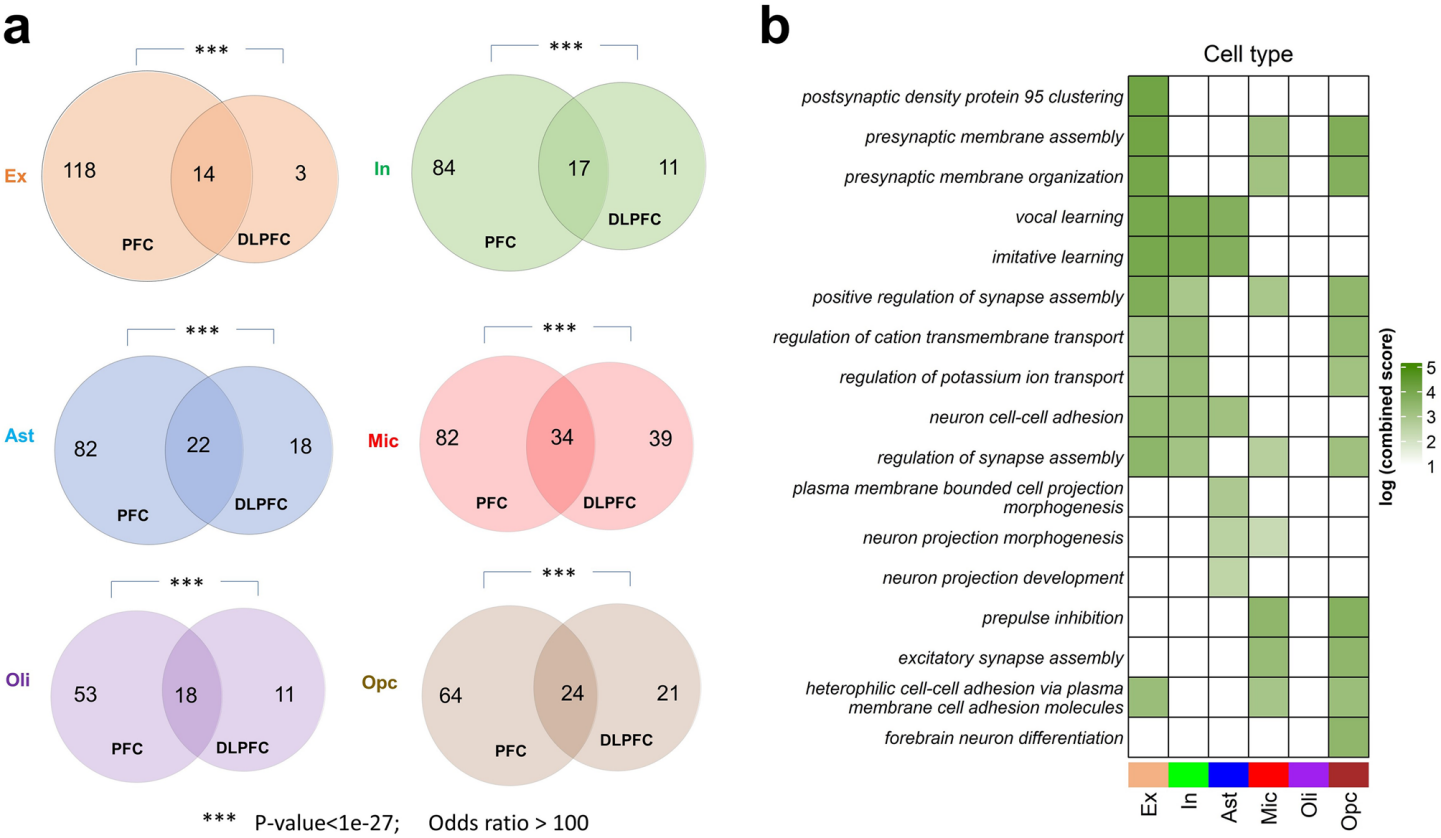
Cross-study validation of RNA velocity differences in neuropathologic AD. (**a**) Venn diagrams for each cell type showing the overlaps between Dataset-1 (prefrontal cortex, PFC) and Dataset-2 (dorsolateral prefrontal cortex, DLPFC) with respect to genes having differential RNA velocity in AD pathology. The sum of all two numbers in any circle represents the number of significant genes in the corresponding dataset. Significance of overlap was estimated with Fisher’s exact test (*p*-value < 0.01; odds ratio > 1). (**b**) GO biological processes and their log-transformed EnrichR combined scores for the overlapping gene sets.

We tested the reproducibility of the observed AD-related differences in RNA velocity in an independent sample. We obtained snRNA-seq data from the dorsolateral prefrontal cortex of another ROSMAP cohort (Sect. “Methods”, “Validation in independent dataset”) comprising 6 cognitively non-impaired individuals with minimal AD pathology and 6 subjects with both pathologic AD and clinical AD dementia^14^. Following preprocessing, we derived 79,472 cells corresponding to the same six cell types under consideration, and 16,844 transcripts (corresponding to 16,829 unique genes) like in our analysis for the first dataset. Out of the 232 genes with significant RNA velocity differences between the two groups in Dataset-2, 129 (i.e., 56%) overlapped with the genes obtained from the initial analysis of the prefrontal cortex (Dataset-1), including 14 in excitatory neurons, 17 inhibitory neurons, 22 in astrocytes, 34 in microglia, 18 in oligodendrocytes and 24 in oligodendrocytes precursor cells (Fig. 4a). The significance of overlap was assessed using Fisher’s exact test (*p*-value < 0.01; odds ratio > 1).

We proceeded to query enriched gene ontology terms (GO) of the significant genes common to the two datasets. We used EnrichR tool^15,16^ to uncover the biological processes associated with these overlapping genes. Importantly, we again found that most of the top biological processes across cell types are associated with neural development and synaptic activities (Fig. 4b). Overall, these findings support the generalizability of the main RNA velocity differences identified, supporting the robustness of this novel technique for the deep molecular characterization and better understanding of AD pathomechanisms.

## Discussion

Here, we use RNA velocity to characterize, for the first time to our knowledge, the dynamical multicellular processes underlying neuropathological AD progression. Unprecedented advances in scRNA-seq have offered a novel way to overcome the poor spatial resolution of bulk tissue mRNA, while enabling the study of cell type-specific changes in AD and related disorders^1,2,4^. However, differences in RNA expression do not completely capture the evolution of the disease continuum or the progressive vulnerability of cells to neurodegeneration. Using snRNA-seq profiled from postmortem brain samples of the prefrontal cortex and dorsolateral prefrontal cortex in two independent studies, we uncovered highly active genes associated with different levels of neuropathology. Importantly, the identified cross-validated dynamic genes are associated with a consistent set of molecular functions linked with neurodegeneration. The results support the validity of the novel RNA velocity concept for achieving a complementary molecular characterization of AD and potentially identifying cell type-specific disease-modifying genetic targets.

We found accelerated cell dynamics in AD subjects compared to controls, which can explain some of the molecular bases of the early changes occurring in AD. A previous study using induced pluripotent stem cells (iPSC) showed that AD brains undergo accelerated neural differentiation that causes early depletion of neural progenitor pools and reduced cell renewal^17^. Further, accelerated cell differentiation may perturb the gene network associated with cell development and synapse organization thereby engendering hyperexcitability and other pathologic cascades. This may as well have implications for the risk of developing dementia as increased cell proliferation of neural progenitor cells in early later and depletion in later life have been linked to *APOE* deficiency^18^. Our study suggests that accelerated cell differentiation occurs across different cell types in AD and offers potential areas for experimental validation.

Our analysis revealed that although RNA velocity is closely related to gene expression, the two quantities may capture different pathological processes. The differentially expressed genes associated with AD pathology differed from those associated with varying RNA velocity. The snRNA velocity related genes are principally involved in cell developmental and synaptic programs while the expression-related genes are mainly implicated in ribosomal and mitochondrial activities. However, most of the snRNA synaptic genes are upregulated, except *EPBH1*, *IL1RAPL1* and *ROBO2* in astrocytes and *PTRD* in excitatory neurons (Supplementary Table 2). Previous studies have shown that astrocytic *EPHB1* and *ROBO2* play vital roles in synapse remodeling and neuronal migration, respectively^19,20^. The interaction of *PTPRD* with *IL1RAPL1* promotes excitatory synapse formation and stabilization, and the downregulating either gene impairs synaptogenesis^21–23^. In addition to β-amyloid and tau related processes, our analysis of snRNA-vel pointed at other potentially altered functions such as voltage-gated cation channels activity and notch signaling which may have dynamic causal roles in AD development but were not detected by the traditional differential expression. Further, we cross-validated the differential RNA velocity analysis in an independent dataset. The overlapping genes between the two datasets are predominantly implicated in biological processes associated with cell development and synapse organization, implying a recurring theme in our results. A profound nexus exists between cell cycle and synaptic activity in AD, and many AD-associated genes are involved in morphoregulation, i.e., the ordered development and arrangement of cells to form synapse through processes such as cell adhesion, cell differentiation, synaptic membrane assembly, ion channel activity, etc. Besides, results from animal studies showed that certain behaviors simultaneously enhance synaptic plasticity and control accelerated cell cycle, thereby protecting against cell death and neurodegeneration^24^.

The RNA velocity metric was designed to capture the dynamic process of cell evolution in the transcriptomic space^5,6^. It was originally applied to infer the developmental states of healthy cells but has found further applications in studying cell proliferation in cancer^9,25^. However, it appears that RNA velocity can also capture dynamic differences associated with severity of AD pathology. Moreover, RNA velocity can be estimated for each cell type at the patient level. Such applications are particularly important for two main reasons. First, prodromal cell changes which occur in AD may be detected before clinical manifestations or the deposition of β-amyloid and tau^26,27^. Second, there are implications for the development of personalized treatment by detecting (and potentially targeting) person-specific contribution of RNA velocity changes to AD neuropathology. We found that most of the biological processes implicated in our study are involved in synapse organization and turnover, a key structural element essential for cognition. Many pathways are also associated with cell developmental processes, the dysfunction of which is linked to neurodegeneration^28^. Thus, our results inform potential therapeutic strategies of targeting substrates of synaptic plasticity, including glutamatergic and cholinergic signaling, and applying cell therapy to enhance cell renewal, differentiation, and proliferation.

This study has some limitations. First, we used single-nucleus RNA sequencing to estimate RNA velocity. Compared to single-cell sequencing, snRNA-seq is more amenable to transcriptomic profiling of postmortem samples because isolated nuclei are intact in frozen tissues^29^. Moreover, dissociating whole cells from the brain is particularly challenging due to the intensity of the required enzymatic activity, which could interfere with cell type recovery and bias the results of downstream analyses ^30^. RNA velocity was originally formulated for scRNA-seq based on the assumption that the rate of RNA degradation is constant across all cells^6^. Importantly, here we showed that there is high concordance between RNA velocities from matched nuclear and whole-cell RNA of microglia, supporting the validity of using snRNA-seq for velocity estimation (see Fig. S1). Furthermore, by using two different RNA velocity methods to achieve a consistent trajectory inference, we demonstrated the robustness of the velocities estimated from single-nucleus data. In line with our observations, a recent study comparing RNA velocity trajectory inferences from scRNA-seq and snRNA-seq showed that each method successfully predicts the transition of retinal resting cells through reactive state to terminal fibrous state. Interestingly, the RNA velocity estimates obtained by combining the two sequencing technologies were consistent with the estimates derived from using either scRNA-seq or snRNA-seq dataset alone. Earlier studies have also successfully applied RNA velocity to single nuclei data to infer biologically meaningful trajectories in mouse embryo and various cell types of the human heart^31,32^. Second, RNA velocity was originally developed to capture rapidly evolving processes with short timescales. Its use in slowly evolving processes, such as neurodegeneration, remains to be validated. However, we believe that the relatively shorter timescale of mRNA transcriptional dynamics may offer a good resolution to capture subtle changes associated with the neuropathological cascade. Despite that the use many of subjects with varied levels of neuropathology allowed us to capture the association between the timescales of transcriptional dynamics and neurodegeneration at the global level, due to experimental limitations we could not directly ascertain the stability of RNA velocities within a subject over different post-mortem intervals. However, we confirmed that the post-mortem sampling intervals between the controls and AD subjects do not differ significantly (Fig. S9), suggesting that our results were not confounded by transcriptional changes induced by death. Importantly, future studies employing multiscale dynamical models of the brain (via gene expression, neuroreceptors, neuroimaging) can also incorporate RNA-vel to better capture the time-resolved complex interactions between multiple biological layers/modalities in neurodegenerative progression^33,34^.

## Methods

### Dataset-1 (Prefrontal cortex)

It includes droplet-based snRNA-seq, neuropathological and clinical data for 48 participants enrolled in the Religious Orders Study (ROS) or the Rush Memory and Aging Project Study (MAP)^35^. The snRNA-seq data was previously generated from the prefrontal cortex (Brodmann area 10) of autopsied brains as described in^1^, and it was downloaded from the Accelerating Medicines Partnership Alzheimer’s Disease knowledge portal (AMP-AD; www.synapse.org, ID syn18485175). All subjects underwent postmortem neuropathologic evaluations, generated in previous ROSMAP studies as described in ^35^ including uniform structured assessment of AD pathology, and other pathologies common in aging and dementia (downloaded from AMP-AD, ID syn3157322; see also Sect. “Correlation with neuropathology”). The 48 subjects (balanced between sexes) comprised 24 with no or low AD-pathology (control group), and 24 with mild to severe AD-pathology (AD group) as determined by β-amyloid burden, neurofibrillary tangles, and cognitive impairment^1^. The subjects were matched for age (medians 87.1 [no pathologic AD, N = 24] and 86.7 [pathologic AD, N = 24]) and years of education (medians of 18 [no pathologic AD] and 19.5 [pathologic AD]). Informed consent was obtained from each participant, and the Religious Orders Study and Rush Memory and Aging Project were approved by an Institutional Review Board (IRB) of Rush University Medical Center. The project was performed in accordance with the Declaration of Helsinki. In addition, subjects signed a repository consent allowing their data to be shared (related documents and requests for data can be obtained at https://www.radc.rush.edu).

The process of isolating the nuclei from the postmortem brain tissues was previously detailed^1^. Briefly, the brain tissue was homogenized at a very low temperature and incubated. The tissue was then filtered and purified with working solutions. The nuclei were separated through spinning at high speed and counted. The sequencing libraries were constructed with the Chromium Single Cell 3′ Reagent Kits v.2 (10 × Genomics) and sequenced with the NextSeq 500/550 High Output v2 kits (150 cycles).

### RNA abundance and cell type identification

Intronic and exonic counts were obtained using kb-python (v0.26.3), a wrapper for kallisto and bustools^36,37^. First, index file of the human genome was generated from the Ensembl human primary reference genome sequence and gene annotation (GRCh38). Then, spliced and unspliced RNA counts were obtained by filtering barcodes with low UMI counts and mapping reads to the index file. The counting process was performed by sequentially running ‘kb ref’, and ‘kb count’ (with filter flag set) commands.

Next, we acquired a previous quality controlled list of genes, cells and cell types^1^. We then looked for shared genes and cells between our filtered counts and the previously reported list. Thus, we had 65,422 cells with 16,844 transcripts (corresponding to 16,829 unique genes). These cells were then assigned to cell types, based on the reported list, as excitatory neurons, inhibitory neurons, astrocytes, microglia, oligodendrocytes, oligodendrocyte precursor cells, endothelial cells, and pericytes. Endothelial and pericyte cells were subsequently excluded because of their very low counts or absence in some subjects.

### RNA velocity estimation

We used scVelo (v0.2.3)^5^ to calculate RNA velocity. First, the cells were pulled together across all subjects, and each cell was normalized by its total size. The normalization was applied to both spliced and unspliced counts. To estimate RNA velocity using the stochastic method, we computed the means and variances of nearest neighbors of cells in principal component analysis (PCA) space. Here, 100 nearest neighbors and 30 principal components were used. Normalization and moments calculation were achieved through ‘pp.normalize_per_cell’ and ‘pp.moments’ commands, respectively. The RNA velocity is then estimated with ‘tl.velocity’ command (setting the mode to ‘stochastic’).

We next sought to validate the estimated RNA velocities by examining the velocity values of the genes driving cell type-specific dynamics. We ran a differential velocity Welch’s t-test with the module ‘scv.tl.rank_velocity_genes’ and obtained the top genes (based on t-value) having cell type-specific differential velocity. We then projected the velocities and expression values of the dynamic genes into t-SNE space to examine their variations across cell types.

### Differential expression and RNA velocity analyses

Cell type-specific gene analysis was performed with Seurat (v4.0.2)^38^ and Presto packages in R. We performed differential expression analysis between the control group and the AD group. Each cell was first normalized by its total count over all genes, scaled by 10,000 and log-transformed. Using the ‘FoldChange’ command in Seurat, we performed Wilcoxon rank-sum test to identify differentially expressed genes at log_2_ (fold change) > 0.25 or < − 0.25. We then used the Presto package (due to its speed) to run 5000 random permutations by randomly reassigning the subjects to either the control or AD group. The U-statistics from the permutations were used to generate null distributions and significance p-values. We identified significant genes after adjusting for multiple testing (q < 0.05, FDR-corrected). To compare differential expression with differential velocity, the procedure was repeated on RNA velocities to identify the dynamic genes driving the velocity difference between the control and AD groups.

### Cell speed and residual velocity estimation

First, the speed of a cell was calculated as the length of its velocity vector. Wilcoxon ranked-sum test was then used to compare the speed between the two groups. Next, the RNA velocities of individual cells were used to derive velocity fields in t-SNE space. For each of the groups (control and AD), we linearly interpolated the velocity fields at the t-SNE coordinates where actual cells are missing to ensure equal number of velocity fields for each group. We then subtracted the velocity fields at same pair of coordinates for the two groups and obtained the z-scores of the norms of these differences. The velocity field difference of those coordinates where the z-score > 1.96 or < − 1.96 are displayed as the residuals between the two groups.

### Correlation with neuropathology

In each cell type and subject, the average RNA velocity across cells was calculated for every gene. The velocities were tested for correlation with four AD neuropathological traits^35^: PHF neurofibrillary tangle density (tangles), neuronal neurofibrillary tangle counts (NFT), overall β-amyloid load (β-amyloid), and neuritic plaque counts (NP). The correlations were adjusted for age, sex, and postmortem interval. Significant genes were chosen based on FWER-corrected *p*-value < 0.001^39^.

### Validation in independent dataset (Dataset-2: Dorsolateral prefrontal cortex)

The droplet-based snRNA-seq data was previously generated from the dorsolateral prefrontal cortex of autopsied brains as described in^14^, and it was downloaded from the Accelerating Medicines Partnership Alzheimer’s Disease knowledge portal (AMP-AD; www.synapse.org, ID syn16780177). The subjects include another 12 sex-matched individuals from Orders Study (ROS) or the Rush Memory and Aging Project Study (MAP)^35^: 6 subjects are cognitively non-impaired with minimal AD pathology and 6 fulfill diagnoses for both pathologic AD and clinical AD dementia.

As previously described^14^, the brain grey matter tissue was homogenized and treated with working solution to separate the nuclei. The isolated nuclei were then counted and filtered. The libraries were constructed and sequenced on the 10X Single Cell RNA-Seq Platform using the Chromium Single Cell 3′ Reagent Kits v2. After obtaining the intronic and exonic counts, genes were selected according to the gene list from Dataset-1 and the cells were filtered using the cell list obtained from the metadata of the previous study^14^. The previously reported cell clusters were used to assign the 79,472 cells to the six cell types under consideration. We calculated the cell type-specific RNA velocities for each subject and used Wilcoxon rank-sum test to identify the genes underlying RNA velocity differences between the minimal AD pathology group and the pathologic/AD dementia group.

We assessed the overlap between the significant differential velocity genes in this dataset and Dataset-1. Fisher’s exact test was used to obtain the significance of overlap (p-value < 0.01 and odds ratio > 1).

### Biological pathway analyses

Biological pathways were identified using EnrichR online tool to query enriched gene ontology (GO) terms^15,16^ from the Gene Ontological Biological Processes 2021. The significant GO terms were selected at an adjusted *p*-value < 0.01 and ranked based on their EnrichR combined scores.

### Comparison between single-cell and single-nucleus RNA velocities (Dataset-3)

We downloaded a previously published dataset from the Gene Expression Omnibus (GEO) (https://www.ncbi.nlm.nih.gov/geo/, GSE135618). The dataset contains matched single cells, fresh nuclei and frozen nuclei obtained from the microglia of two subjects^40^. After preprocessing, we obtained 2988 cells, 4892 fresh nuclei, and 4019 frozen nuclei from one subject; and 3485 cells, 2593 fresh nuclei, and 5527 frozen nuclei from the other subject. For each of the subjects, we estimated the RNA velocities across the three modalities (single cell, fresh nuclei, and frozen nuclei) and performed within-subject comparisons of the velocity estimates.

### Data visualization

Visualizations were performed using scVelo (v.0.2.3)^5^, ComplexHeatMap (v.2.6.2)^41^, g:Profiler^42^, Cytoscape App (3.9.1)^43^, and STRING (v.12.0)^44^.

### Supplementary Information

Supplementary Figures.

Supplementary Table 1.

Supplementary Table 2.

Supplementary Table 3.

Supplementary Table 4.

Supplementary Table 5.

## Data Availability

Dataset-1 snRNA-seq and metadata are available at the AMP-AD portal (https://www.synapse.org, IDs syn18485175 and syn3157322). Dataset-2 snRNA-seq and metadata can also be downloaded from the AMP-AD portal (https://www.synapse.org, ID syn16780177). The raw scRNA-seq of Dataset-3 are available on the GEO (https://www.ncbi.nlm.nih.gov/geo/, accession code GSE135618). The data on GEO is freely accessible without registration while datasets on Synapse are available under controlled use conditions to ensure anonymity of the study participants. Hence, data use agreement and registration are required to access Dataset-1 and Dataset-2. ROSMAP data can be requested at https://www.radc.rush.edu.

## Code availability

scVelo (v.0.2.3) and veloVI (v.0.3.0) are downloadable as python packages (see https://scvelo.readthedocs.io/installation/ and https://velovi.readthedocs.io/en/latest/). The codes used for the analyses will be available with article publication at https://neuropm-lab.com/other-pipelines.
