# Supplementary material for: Single-nucleus RNA velocity reveals critical synaptic and cell-cycle dysregulations in neuropathologically confirmed Alzheimer’s disease
Source: Sci Rep. 2024 Mar 27;14:7269. doi: 10.1038/s41598-024-57918-x (PMC10973452; doi:10.1038/s41598-024-57918-x)
Supplement: Supplementary file 1 — Supplementary Figures. [file 41598_2024_57918_MOESM1_ESM.doc]

**Single-nucleus RNA velocity reveals critical synaptic and cell-cycle dysregulations in neuropathologically confirmed Alzheimer’s disease**

Quadri Adewale^1,2,3^, Ahmed F Khan ^1,2,3^, David A. Bennett^4,5^ and Yasser Iturria-Medina^1,2,3^*

^1^ Neurology and Neurosurgery Department, Montreal Neurological Institute, McGill Univ., Montreal, Canada.

^2^ McConnell Brain Imaging Centre, Montreal Neurological Institute, McGill Univ., Montreal, Canada.

^3^ Ludmer Centre for Neuroinformatics and Mental Health, McGill Univ., Montreal, Canada.

^4^ Rush Alzheimer's Disease Center, Rush University Medical Center, Chicago, IL, USA.

^5^ Department of Neurological Sciences, Rush University Medical Center, Chicago, IL, USA.

* Correspondence to: Y I-M, 3801 University Street, room NW312, Montreal Neurological Institute, McGill University, Montreal, Canada H3A 2B4. Email: [yasser.iturriamedina@mcgill.ca](mailto:yasser.iturriamedina@mcgill.ca)

**Supplementary Figures**

**Validation of the Correspondence Between RNA scRNA-seq and snRNA-seq in Microglia**
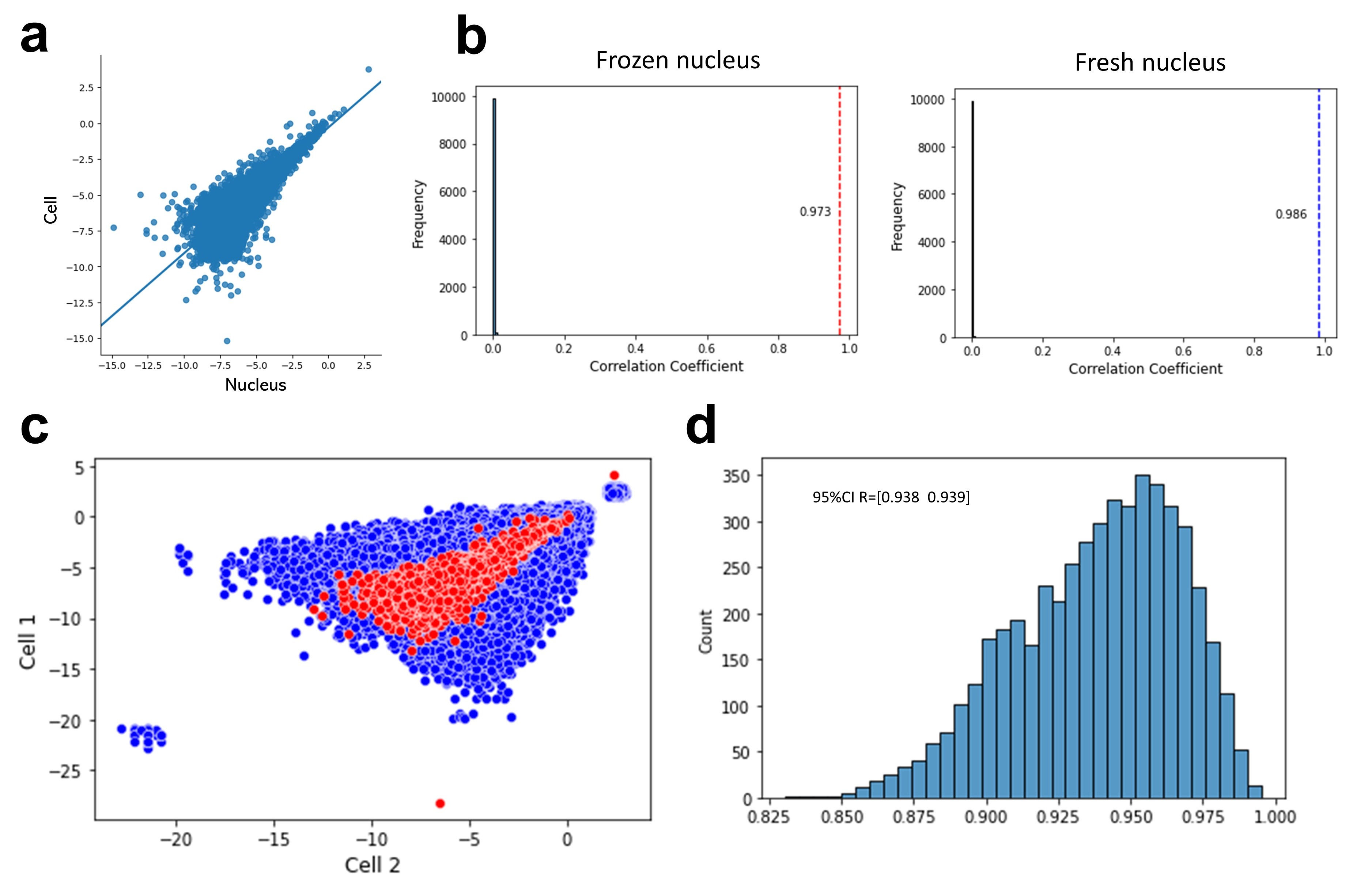


Figure S1 | Validation of the correspondence between RNA velocities of single-cell and single-nucleus sequencing dataset from microglia of same subjects. a) Scatter plot (in log scale) showing the linear concordance between velocities calculated in cells and nuclei for a subject. Each dot represents an independent gene. b) Left: null distribution of Pearson’s correlation coefficient between scRNA and frozen snRNA velocities from 5000 permutations. The actual correlation (0.973) is shown in red dotted line. Right: null distribution of Pearson’s correlation coefficient between scRNA and fresh snRNA velocities from 5000 permutations. The actual correlation (0.986) is shown in blue dotted line. The second subject (not shown) had correlations of 0.936 and 0.944 in frozen and fresh nuclei, respectively. The high and significant correlations suggest that nuclei RNA can be used to estimate RNA velocities in place of whole-cell RNA. c) Overlay of cell-cell (in blue) and cell-nucleus (in red) RNA velocity correlations. First, a pair of cells is selected at random and the velocities of the genes in the cells are plotted against each other. The random selection is repeated 5000 times to show the variabilities in RNA velocity correlations between single cells. The initial cell-nucleus correlation from A) is then overlayed on the cell-cell correlation plot. The variabilities in cell-nucleus correlation of RNA velocity are observed present in correlations between single cells. D) The correlation distribution between the RNA velocities of any random pair of whole cells. The 95% confidence interval is shown in the plot.

**Comparison of RNA Velocity Trajectory Inference Between scVelo and veloVI**


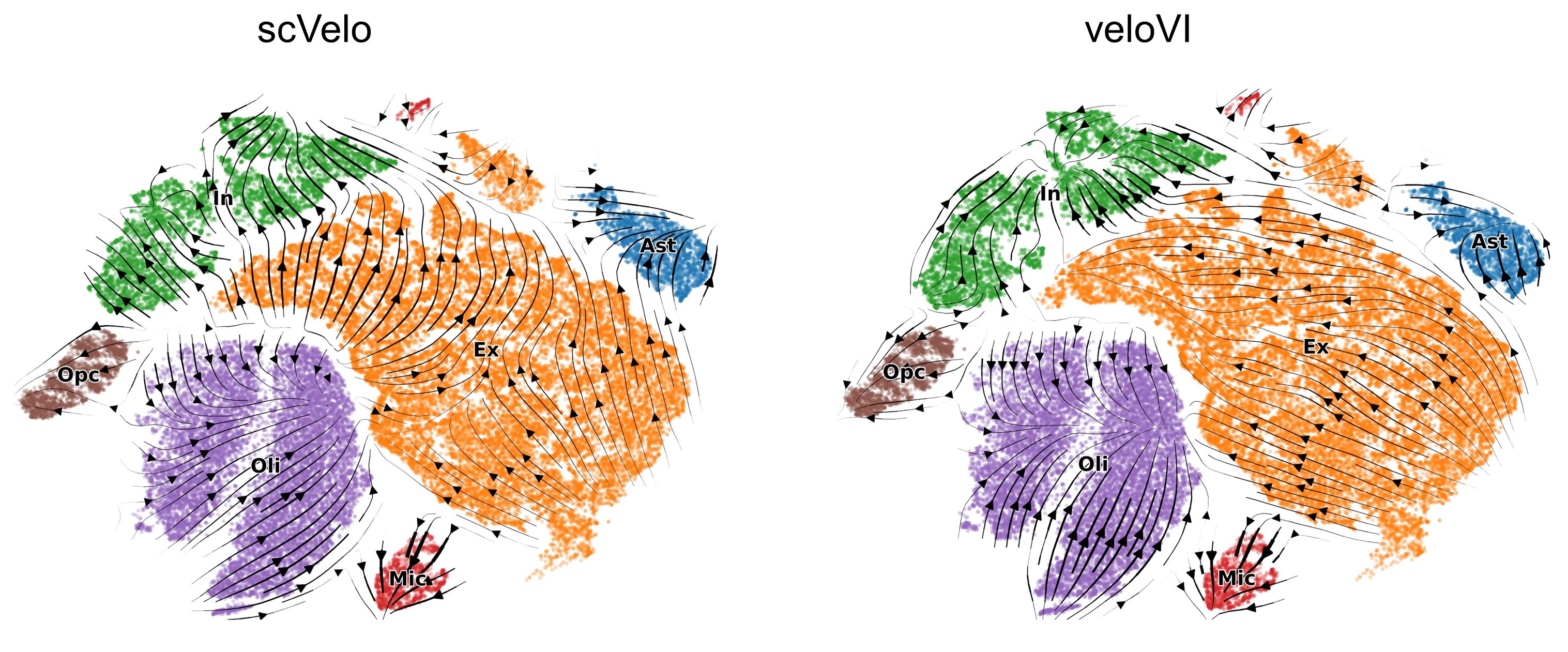
Figure S2 | Comparison of single-nucleus RNA velocity trajectory predictions between scVelo and veloVI. Velocities were projected on the cells across 48 subjects. Inferred directionality was concordant across cell types, expect for minor differences in excitatory neurons.

**Effect of Age on Differential RNA Velocity Between Controls and AD-Pathology Subjects**


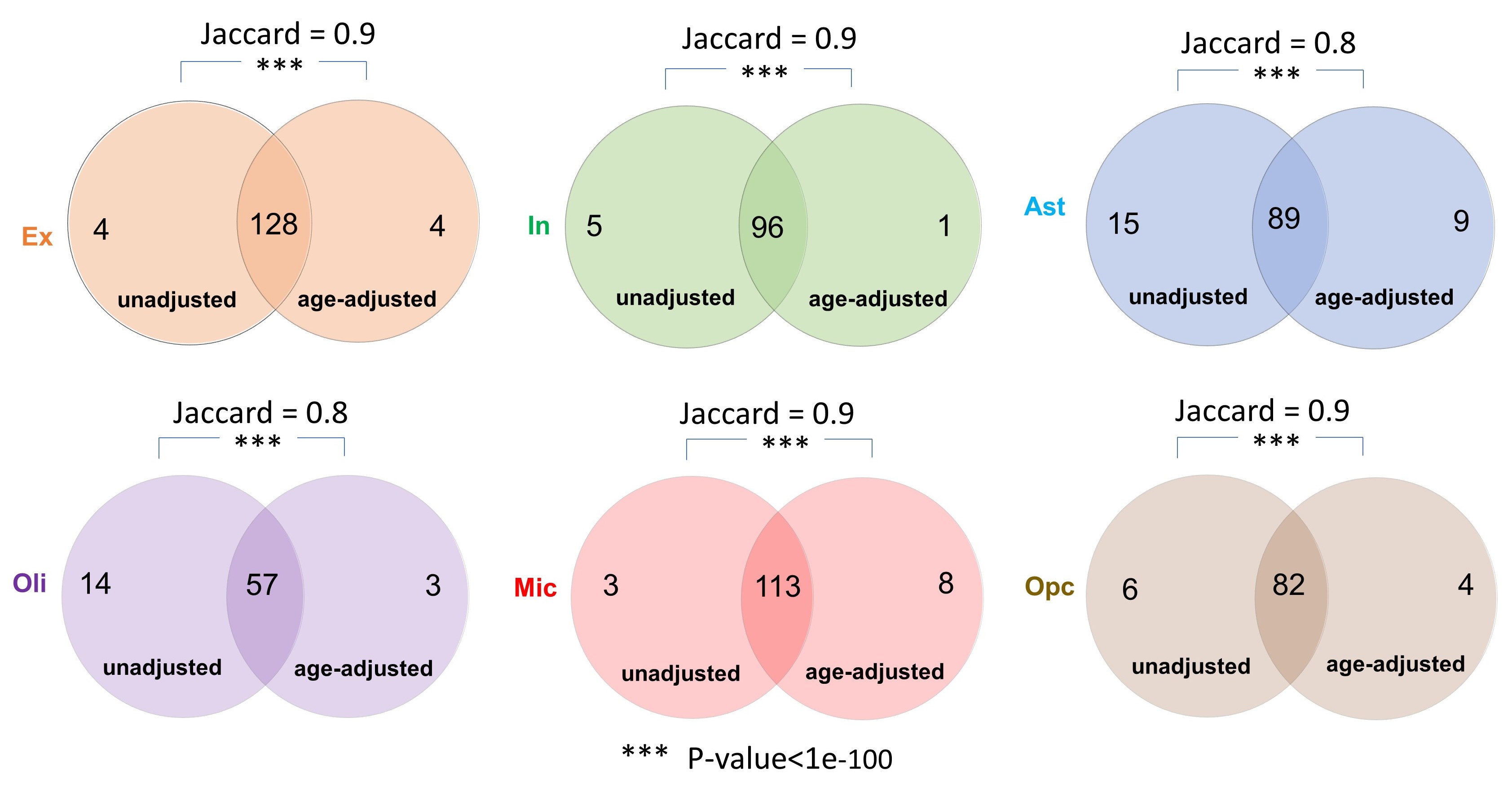


Figure S3 | Comparison of group differences in RNA velocity between controls and AD-pathology subjects before and after correcting for age. Jaccard similarity between the unadjusted and age-corrected analyses demonstrates the absence of confounding effect, possibly because the subjects were matched for age.

**Differential RNA Velocities of Neuronal Cells Are Not Biased by Gene Length**


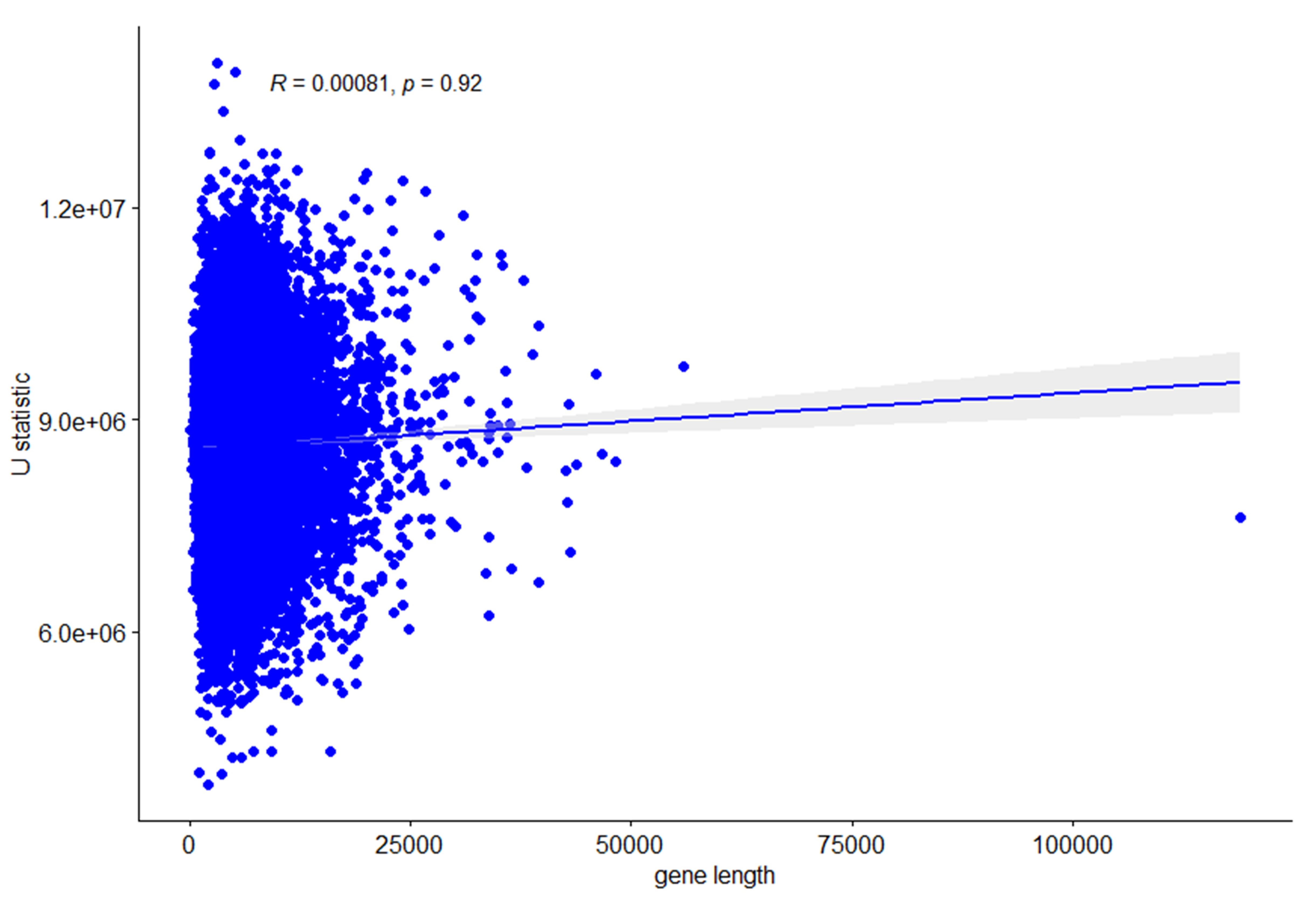


Figure S4 | Correlation between gene length and Wilcoxon rank sum test U-statistic (from differential velocity analysis between AD and control groups) in inhibitory neurons. As there is a tendency for RNA velocities in neurons to be biased towards gene length, we investigated whether the results of differential velocity analyses we performed were influenced by gene length. We found that the correlation between gene length and U-statistic is very minimal and insignificant, supporting that the significant genes identified are not biased by gene length.

**Protein-Protein Interactions of Genes Associated with Ion Channel Activity and Synaptic Processes in Excitatory Neurons**


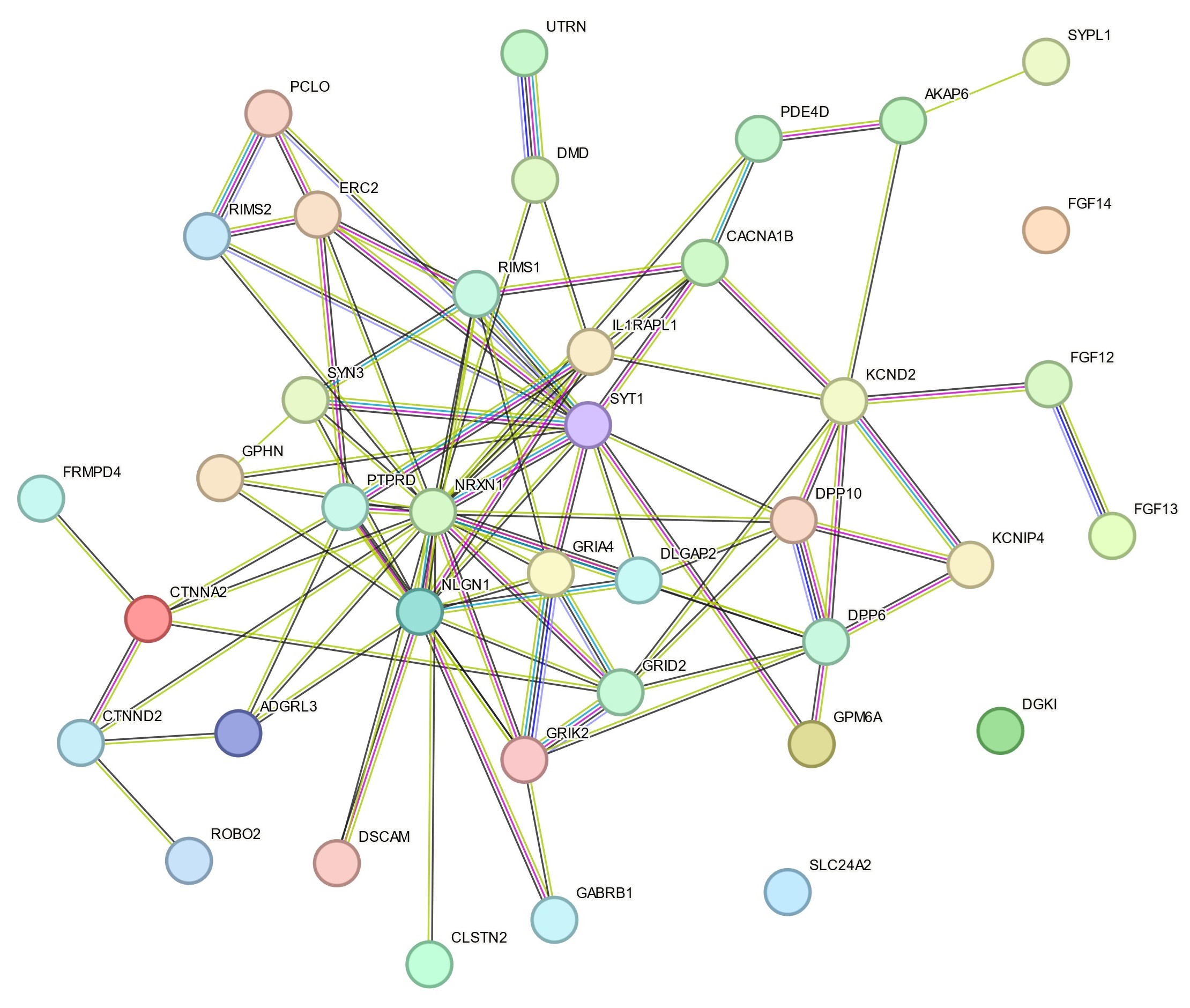


Figure S5 | Protein-protein interaction network of the differential velocity genes implicated in synaptic and ion channel activities in excitatory neurons. PPI network was retrieved from STRING database.

**Changes in Transcriptional Kinetics with AD Progression**

**
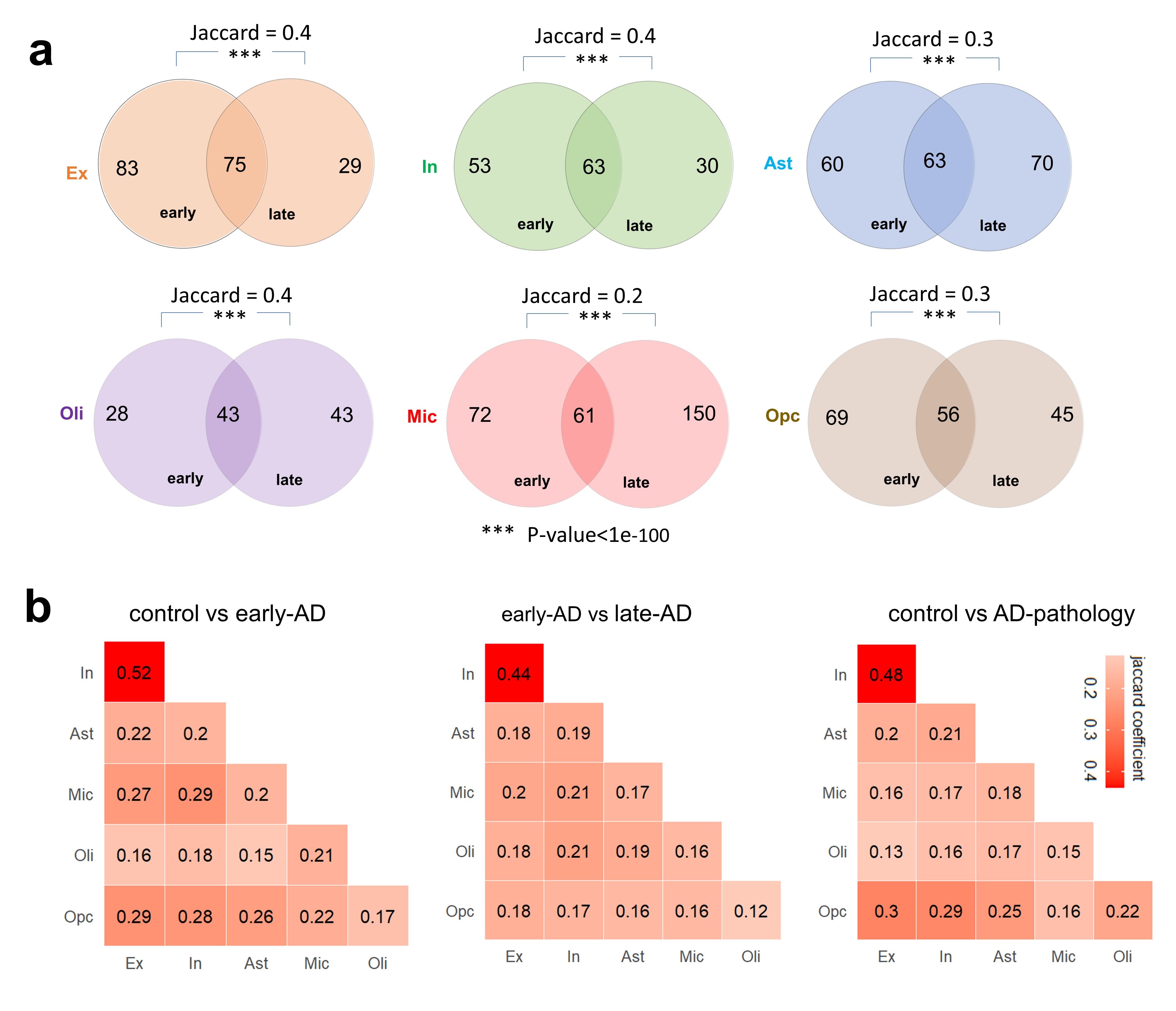
**

Figure S6 | Changes in RNA velocity with disease progression. a) Comparison of the number of genes with differential RNA velocity between “control versus early-AD analysis (left circle)” and “control versus late-AD analysis (right circle)”. b) Cell-specificity of changes in transcriptional dynamics with disease progression. First, isolated nuclei were compared between control and AD-pathology subjects to determine genes with differential RNA velocity (right). Then, the level of overlap between differential velocity genes across cell types is quantified via Jaccard similarity. The analyses were repeated for “early- versus late-AD individuals” (middle); and “control versus early-AD subjects” (left).

**Comparison of Enriched Pathways in Male and Female Subjects**

**
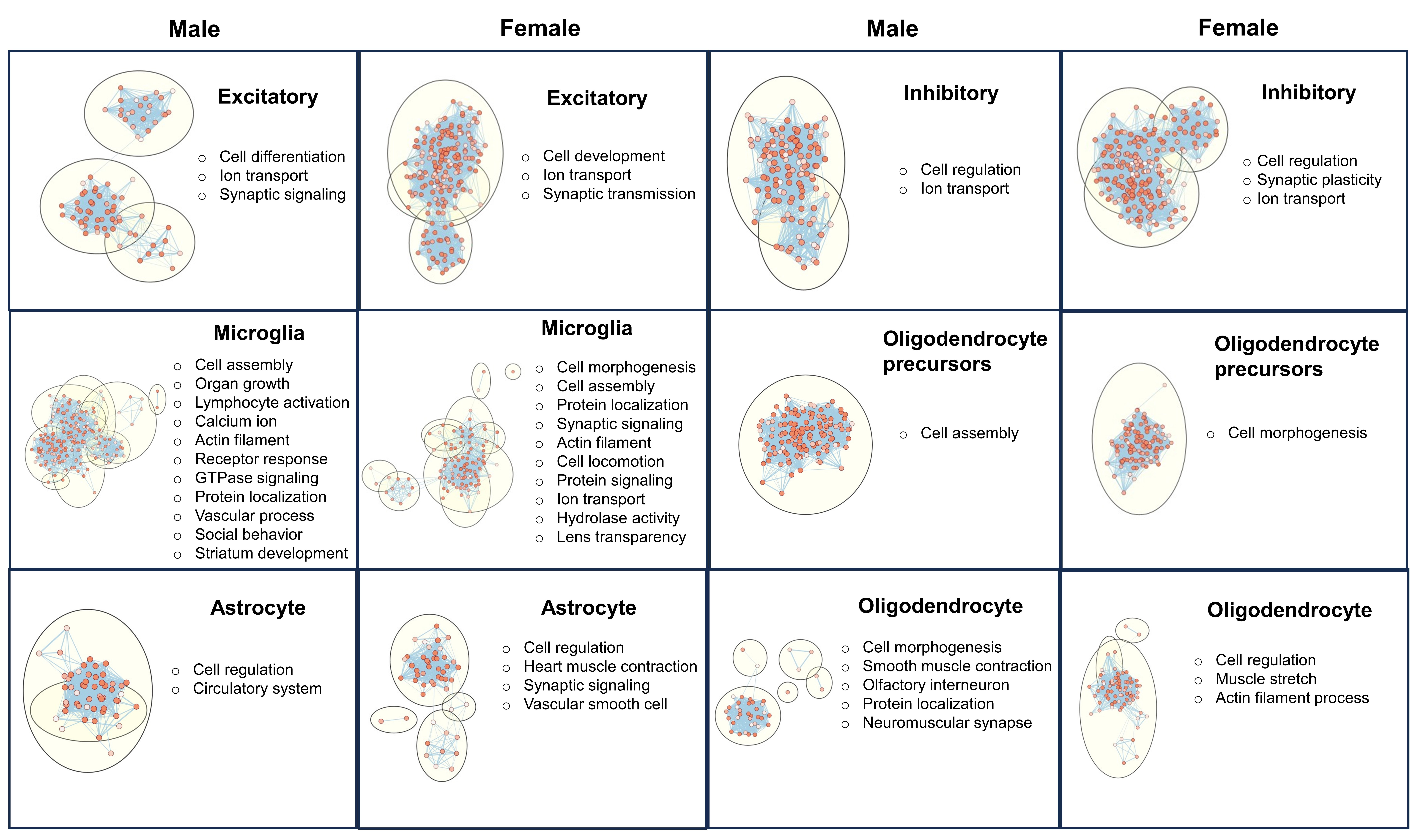
**

Figure S7 | Enriched disease pathway networks in male and female across six cell types. GO biological processes derived from differential velocity genes between controls and AD-pathology subjects are clustered into biological themes (adjusted p-value < 0.01). The biological themes are listed in increasing order of cluster size.

**Inter-Cell Overlap of Dynamic Genes Correlated with AD Pathology**


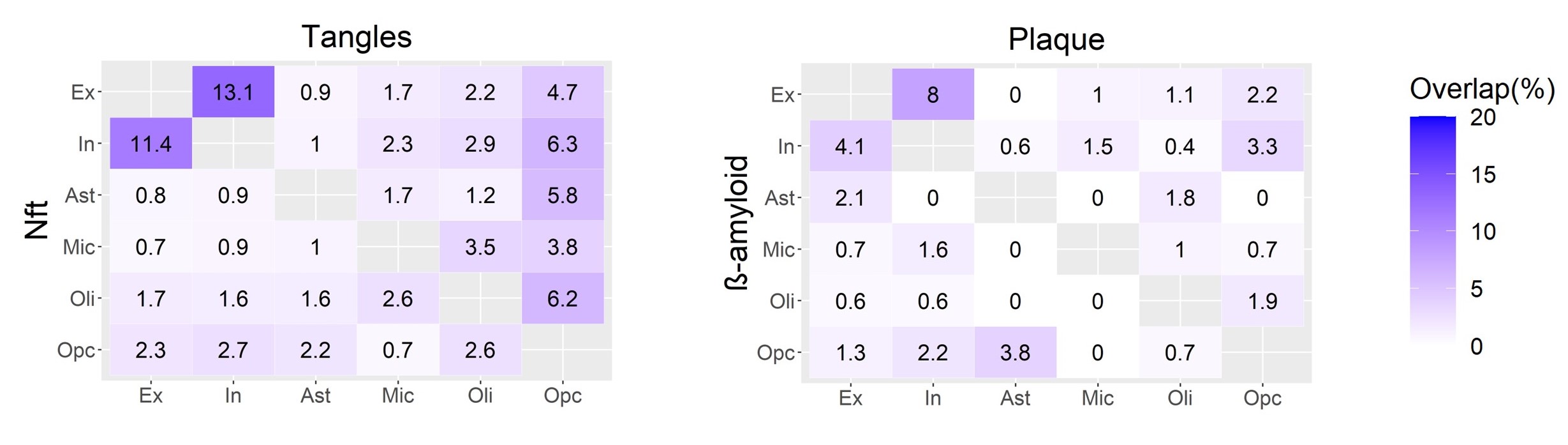


Figure S8 | Percentage overlap of dynamic genes associated with AD pathology in each cell type. The percentage is obtained by expressing the number of genes common to any two cell types as a fraction of the total number of unique genes in the two cell types. Two neuropathological variables are visualized simultaneously on the same plot. Only excitatory and inhibitory neurons presented a relatively high overlapping (up to 13%) in significant genes associated with the different AD neuropathological traits. The other cell types substantially differed in gene-specific changes across the phenotypes.

**Differential Velocity is Not Confounded by Post-Mortem Interval**


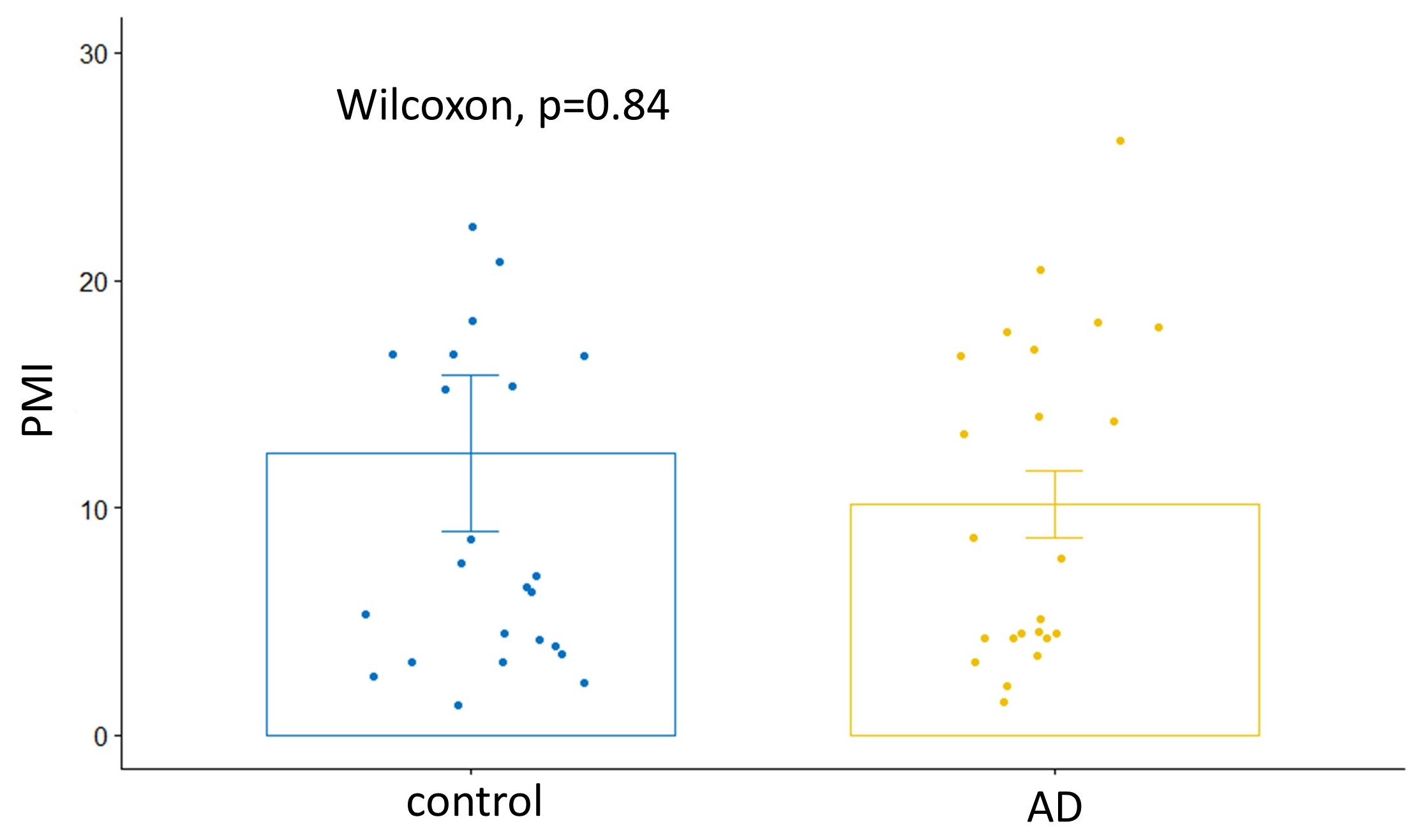


Figure S9 | Test for difference in post-mortem interval between the control and AD groups. Wilcoxon rank-sum test shows that there is no significant difference in the post-mortem sampling intervals, indicating that the differences captured between the two groups is related to pathological process.
